# Supplementary material for: Assessment of the Impact of Lower Urinary Tract Dysfunction on Quality of Life in Multiple Sclerosis Patients in Saudi Arabia—A Cross-Sectional Study
Source: Healthcare (Basel). 2023 Oct 9;11(19):2694. doi: 10.3390/healthcare11192694 (PMC10572286; doi:10.3390/healthcare11192694)
Supplement: Supplementary file 1 [file healthcare-11-02694-s001.zip › healthcare-2578749-supplementary.pdf]

Table S1. The LURN questionnaire scores for the participants.

Questionnaire		Frequency	patient		Gender				P – value*
					Females		Males		
			Count	%	Count	%	Count	%	
	1. In the past 7 days, how often did you completely lose control of your bladder?	never	183	42.80%	122	45.20%	61	38.60%	0.325
		a few times	133	31.10%	85	31.50%	48	30.40%	
		about half the times	34	7.90%	18	6.70%	16	10.10%	
		most of the time	49	11.40%	26	9.60%	23	14.60%	
		every time	29	6.80%	19	7.00%	10	6.30%	
	2. In the past 7 days, how often did you leak urine or wet a pad after feeling a sudden need to urinate?	never	228	53.30%	149	55.20%	79	50.00%	0.2
		a few times	128	29.90%	82	30.40%	46	29.10%	
		about half the times	28	6.50%	13	4.80%	15	9.50%	
		most of the time	32	7.50%	17	6.30%	15	9.50%	
		every time	12	2.80%	9	3.30%	3	1.90%	
	3. In the past 7 days, how often did you leak urine or wet a pad while laughing, sneezing, or coughing?	never	279	65.20%	174	64.40%	105	66.50%	0.434
		a few times	104	24.30%	67	24.80%	37	23.40%	
		about half the times	20	4.70%	10	3.70%	10	6.30%	
		most of the time	18	4.20%	13	4.80%	5	3.20%	
		every time	7	1.60%	6	2.20%	1	0.60%	
Incontinence	4. In the past 7 days, how often did you leak urine or wet a pad when doing physical activities, such as exercising or lifting a heavy object?	never	292	68.20%	188	69.60%	104	65.80%	0.534
		a few times	99	23.10%	62	23.00%	37	23.40%	
		about half the times	19	4.40%	11	4.10%	8	5.10%	
		most of the time	14	3.30%	6	2.20%	8	5.10%	
		every time	4	0.90%	3	1.10%	1	0.60%	
	5. In the past 7 days, how often did walking at your usual speed cause you to leak urine or wet a pad?	never	292	68.20%	187	69.30%	105	66.50%	0.54
		a few times	95	22.20%	56	20.70%	39	24.70%	
		about half the times	20	4.70%	11	4.10%	9	5.70%	
		most of the time	15	3.50%	11	4.10%	4	2.50%	
		every time	6	1.40%	5	1.90%	1	0.60%	
	6. In the past 7 days, how often did you leak urine during the night, including wetting a pad or the bed?	never	337	78.70%	220	81.50%	117	74.10%	0.15
		a few nights	61	14.30%	37	13.70%	24	15.20%	
		about half the nights	12	2.80%	4	1.50%	8	5.10%	
		most nights	14	3.30%	7	2.60%	7	4.40%	
		every night	4	0.90%	2	0.70%	2	1.30%	
	7. In the past 7 days, how often did you have pain or discomfort in your bladder while it was filling?	never	150	35.00%	103	38.10%	47	29.70%	0.354
		a few times	150	35.00%	91	33.70%	59	37.30%	
		about half the times	38	8.90%	23	8.50%	15	9.50%	
		most of the time	65	15.20%	36	13.30%	29	18.40%	
		every time	25	5.80%	17	6.30%	8	5.10%	
Bladder pain	8. In the past 7 days, how often did you have pain or discomfort in your bladder when it was full?	never	132	30.80%	87	32.20%	45	28.50%	0.316
		a few times	156	36.40%	103	38.10%	53	33.50%	
		about half the times	52	12.10%	28	10.40%	24	15.20%	
		most of the time	63	14.70%	35	13.00%	28	17.70%	
		every time	25	5.80%	17	6.30%	8	5.10%	
	9. In the past 7 days, how often did you have pain or discomfort while urinating?	never	204	47.70%	143	53.00%	61	38.60%	0.000326
		a few times	132	30.80%	87	32.20%	45	28.50%	
		about half the times	38	8.90%	15	5.60%	23	14.60%	

		most of the time	34	7.90%	17	6.30%	17	10.80%	
		every time	20	4.70%	8	3.00%	12	7.60%	
	10. In the past 7 days, how often did you have pain or discomfort right after you had finished urinating?	never	250	58.40%	171	63.30%	79	50.00%	
		a few times	97	22.70%	59	21.90%	38	24.10%	
		about half the times	32	7.50%	14	5.20%	18	11.40%	0.033
		most of the time	34	7.90%	18	6.70%	16	10.10%	
		every time	15	3.50%	8	3.00%	7	4.40%	
	11. In the past 7 days, how often did you have to push when urinating?	never	169	39.50%	118	43.70%	51	32.30%	
		a few times	144	33.60%	87	32.20%	57	36.10%	
		about half the times	47	11.00%	28	10.40%	19	12.00%	0.17
		most of the time	48	11.20%	27	10.00%	21	13.30%	
		every time	20	4.70%	10	3.70%	10	6.30%	
	12. In the past 7 days, how often did you have a delay before you started to urinate?	never	175	40.90%	133	49.30%	42	26.60%	
		a few times	141	32.90%	84	31.10%	57	36.10%	
		about half the times	40	9.30%	20	7.40%	20	12.70%	0.00004
		most of the time	49	11.40%	23	8.50%	26	16.50%	
		every time	23	5.40%	10	3.70%	13	8.20%	
	13. In the past 7 days, once you started urinating, how often did your urine flow stop and start again?	never	183	42.80%	128	47.40%	55	34.80%	
		a few times	134	31.30%	84	31.10%	50	31.60%	
Voiding difficulty		about half the times	45	10.50%	32	11.90%	13	8.20%	0.000301
		most of the time	43	10.00%	16	5.90%	27	17.10%	
		every time	23	5.40%	10	3.70%	13	8.20%	
	14. In the past 7 days, how often was your urine flow slow or weak?	never	165	38.60%	115	42.60%	50	31.60%	
		a few times	147	34.30%	99	36.70%	48	30.40%	
		about half the times	50	11.70%	26	9.60%	24	15.20%	0.003
		most of the time	39	9.10%	19	7.00%	20	12.70%	
		every time	27	6.30%	11	4.10%	16	10.10%	
	15. In the past 7 days, how often did you have a trickle or dribble at the end of your urine flow?	never	143	33.40%	106	39.30%	37	23.40%	
		a few times	131	30.60%	85	31.50%	46	29.10%	
		about half the times	48	11.20%	30	11.10%	18	11.40%	0.000096
		most of the time	59	13.80%	23	8.50%	36	22.80%	
		every time	47	11.00%	26	9.60%	21	13.30%	
	16. In the past 7 days, how often did you feel a sudden need to urinate?	never	124	29.00%	90	33.30%	34	21.50%	
		a few times	93	21.70%	66	24.40%	27	17.10%	
		about half the times	105	24.50%	53	19.60%	52	32.90%	0.003
		most of the time	72	16.80%	40	14.80%	32	20.30%	
		every time	34	7.90%	21	7.80%	13	8.20%	
	17. In the past 7 days, how often did you have a sudden need to rush to urinate for fear of leaking urine?	never	160	37.40%	107	39.60%	53	33.50%	
		a few times	71	16.60%	48	17.80%	23	14.60%	
Urgency		about half the times	101	23.60%	58	21.50%	43	27.20%	0.193
		most of the time	72	16.80%	46	17.00%	26	16.50%	
		every time	24	5.60%	11	4.10%	13	8.20%	
	18. In the past 7 days, once you noticed the need to urinate, how difficult was it to wait more than a few minutes?	Not difficult	139	32.50%	99	36.70%	40	25.30%	
		A little difficult	84	19.60%	51	18.90%	33	20.90%	
		somewhat difficult	138	32.20%	83	30.70%	55	34.80%	0.131
		very difficult	50	11.70%	29	10.70%	21	13.30%	
		unable to wait	17	4.00%	8	3.00%	9	5.70%	
Nocturia		None	104	24.30%	69	25.60%	35	22.20%	0.47
		1 time	137	32.00%	85	31.50%	52	32.90%	

	19. In the past 7 days, during a typical night, how many times did you wake up and urinate?	2 - 3 times	127	29.70%	83	30.70%	44	27.80%	
		more than 3 times	60	14.00%	33	12.20%	27	17.10%	
	20. In the past 7 days, how often did you wake up at least once during the night because you had to urinate?	never	110	25.70%	69	25.60%	41	25.90%	0.159
		a few nights	138	32.20%	97	35.90%	41	25.90%	
		about half the nights	123	28.70%	74	27.40%	49	31.00%	
		most nights	34	7.90%	19	7.00%	15	9.50%	
		every night	23	5.40%	11	4.10%	12	7.60%	
	21. In the past 7 days, during waking hours, how many times did you typically urinate?	3 or fewer times a day	157	36.70%	104	38.50%	53	33.50%	0.212
		4-7 times a day	189	44.20%	109	40.40%	80	50.60%	
		8-10 times a day	59	13.80%	41	15.20%	18	11.40%	
		11 or more times a day	23	5.40%	16	5.90%	7	4.40%	
	22. In the past 7 days, during a typical day, how much time typically passed between urinations?	More than 6 hours	66	15.40%	33	12.20%	33	20.90%	0.176
		5-6 hours	79	18.40%	58	21.10%	21	13.30%	
		3-4 hours	247	57.60%	157	58.10%	90	57.00%	
		1-2 hours	0	0.00%	0	0.00%	0	0.00%	
		Less than 1 hour	37	8.60%	23	8.50%	14	8.90%	
	23. In the past 7 days, how would you describe your typical urge to urinate when you woke up during the night?	No urge	128	29.90%	77	28.50%	51	32.30%	0.452
		Mild urg	109	25.50%	64	23.70%	44	27.80%	
		Moderate urge	115	26.90%	76	28.10%	40	25.30%	
		Strong urge	76	17.80%	53	19.60%	23	14.60%	
	24. In the past 7 days, did you have a constant need to urinate that did not go away?	Yes	244	56.60%	160	59.30%	84	53.20%	0.219
		No	184	43.40%	110	40.70%	74	46.80%	
	25. In the past 7 days, how often did you feel that your bladder was not completely empty after urination?	never	120	28.00%	64	23.70%	57	36.10%	0.008
		a few times	182	42.50%	116	43.00%	66	41.80%	
		about half the times	39	9.30%	23	8.50%	16	10.10%	
		most of the time	58	13.70%	43	15.90%	15	9.50%	
		every time	28	6.50%	24	8.90%	4	2.50%	
Post micturition	26. In the past 7 days, how often did you dribble urine just after zipping your pants or pulling up your underwear?	never	238	55.60%	148	54.80%	90	57.00%	2.04E-07
		a few times	123	28.70%	74	27.40%	49	31.00%	
		about half the times	28	6.50%	15	5.60%	13	8.20%	
		most of the time	26	6.10%	24	8.90%	2	1.30%	
		every time	13	3.00%	9	3.30%	4	2.50%	
	27. In the past 7 days, how often did you have spraying or change in direction (men) / splitting (women) of your urine stream?	never	200	46.70%	130	48.10%	70	44.30%	0.946
		a few times	132	30.80%	82	30.40%	50	31.60%	
		about half the times	37	8.60%	23	8.50%	14	8.90%	
		most of the time	37	8.60%	22	8.10%	15	9.50%	
		every time	22	5.10%	13	4.80%	9	5.70%	
Urinary symptoms	28. In the past 7 days, how bothered were you by urinary symptoms?	Not at all bothered	123	28.70%	76	28.10%	47	29.70%	0.041
		Somewhat bothered	183	42.80%	106	39.30%	77	48.70%	
		Very bothered	59	13.80%	39	14.40%	20	12.70%	
		Extremely bothered	63	14.70%	49	18.10%	14	8.90%	

Table S2. The WHOQOL-BREF questionnaire scores for the participants.

Questionnaire		Frequency	patient		Gender				P - value*
					Females		Males		
			Count	%	Count	%	Count	%	
1. How would you rate your quality of life?		Very poor	15	3.5%	9	3.3%	6	3.8%	0.043
		Poor	38	8.9%	23	8.5%	15	9.5%	
		Neither poor nor good	186	43.5%	104	38.5%	82	51.9%	
		Good	132	30.8%	96	35.6%	36	22.8%	
		Very good	57	13.3%	38	14.1%	19	12.0%	
2. How satisfied are you with your health?		Very dissatisfied	25	5.8%	16	5.9%	9	5.7%	0.537
		Dissatisfied	38	8.9%	19	7.0%	19	12.0%	
		Neither satisfied nor dissatisfied	136	31.8%	87	32.2%	49	31.0%	
		Satisfied	162	37.9%	104	38.5%	58	36.7%	
		Very satisfied	67	15.7%	44	16.3%	23	14.6%	
Physiological	3.To what extent do you feel that physical pain prevents you from doing what you need to do?	Not at all	37	8.6%	27	10.0%	10	6.3%	0.612
		A little	73	17.1%	45	16.7%	28	17.7%	
		A moderate amount	193	45.1%	123	45.6%	70	44.3%	
		Very much	19	4.4%	10	3.7%	9	5.7%	
		An extreme	106	24.8%	65	24.1%	41	25.9%	
	4. How much do you need any medical treatment to function in your daily life?	Not at all	67	15.7%	36	13.3%	31	19.6%	0.007
		A little	69	16.1%	33	12.2%	36	22.8%	
		A moderate amount	136	31.8%	91	33.7%	45	28.5%	
		Very much	24	5.6%	17	6.3%	7	4.4%	
		An extreme	132	30.8%	93	34.4%	39	24.7%	
	10. Do you have enough energy for everyday life?	Not at all	36	8.4%	25	9.3%	11	7.0%	0.090
		A little	102	23.8%	61	22.6%	41	25.9%	
		Moderately	153	35.7%	103	38.1%	50	31.6%	
		Mostly	81	18.9%	54	20.0%	27	17.1%	
		Completely	56	13.1%	27	10.0%	29	18.4%	
	15. How well are you able to get around?	Very poor	32	7.5%	19	7.0%	13	8.2%	0.008
		Poor	50	11.7%	24	8.9%	26	16.5%	
		Neither poor nor good	90	21.0%	48	17.8%	42	26.6%	
		Good	116	27.1%	81	30.0%	35	22.2%	
		Very good	140	32.7%	98	36.3%	42	26.6%	
	16. How satisfied are you with your sleep?	Very dissatisfied	60	14.0%	37	13.7%	23	14.6%	0.429
		Dissatisfied	70	16.4%	41	15.2%	29	18.4%	
		Neither satisfied nor dissatisfied	132	30.8%	78	28.9%	54	34.2%	
		Satisfied	126	29.4%	86	31.9%	40	25.3%	
		Very satisfied	40	9.3%	28	10.4%	12	7.6%	
	17. How satisfied are you with your ability to perform	Very dissatisfied	43	10.0%	28	10.4%	15	9.5%	0.971
		Dissatisfied	72	16.8%	44	16.3%	28	17.7%	
		Neither satisfied nor dissatisfied	126	29.4%	78	28.9%	48	30.4%	

	your daily living activities?	Satisfied	129	30.1%	84	31.1%	45	28.5%	
		Very satisfied	58	13.6%	36	13.3%	22	13.9%	
	18. How satisfied are you with your capacity for work?	Very dissatisfied	61	14.3%	36	13.3%	25	15.8%	0.456
		Dissatisfied	65	15.2%	37	13.7%	28	17.7%	
		Neither satisfied nor dissatisfied	121	28.3%	83	30.7%	38	24.1%	
		Satisfied	125	29.2%	81	30.0%	44	27.8%	
		Very satisfied	56	13.1%	33	12.2%	23	14.6%	
	5. How much do you enjoy life?	Not at all	43	10.0%	27	10.0%	16	10.1%	0.955
		A little	6	1.4%	3	1.1%	3	1.9%	
		A moderate amount	179	41.8%	113	41.9%	66	41.8%	
		Very much	92	21.5%	60	22.2%	32	20.3%	
		An extreme amount	108	25.2%	67	%24.8	41	25.9%	
	6. To what extent do you feel your life to be meaningful?	Not at all	54	12.6%	31	11.5%	23	14.6%	0.595
		A little	12	2.8%	8	3.0%	4	2.5%	
		A moderate amount	108	25.2%	69	25.6%	39	24.7%	
		Very much	97	22.7%	67	24.8%	30	19.0%	
		An extreme amount	157	36.7%	95	35.2%	62	39.2%	
	7. How well are you able to concentrate?	Not at all	51	11.9%	28	10.4%	23	14.6%	0.053
		A little	11	2.6%	7	2.6%	4	2.5%	
		A moderate amount	214	50.0%	137	50.7%	77	48.7%	
		Very much	93	21.7%	68	25.2%	25	15.8%	
		extremely	59	13.8%	30	11.1%	29	18.4%	
Psychological	11. Are you able to accept your bodily appearance?	Not at all	38	8.9%	28	10.4%	10	6.3%	0.658
		A little	62	14.5%	37	13.7%	25	15.8%	
		Moderately	73	17.1%	44	16.3%	29	18.4%	
		Mostly	93	21.7%	58	21.5%	35	22.2%	
		Completely	162	37.9%	103	38.1%	59	37.3%	
	19. How satisfied are you with yourself?	Very dissatisfied	38	8.9%	31	11.5%	7	4.4%	0.1
		Dissatisfied	60	14.0%	35	13.0%	25	15.8%	
		Neither satisfied nor dissatisfied	89	20.8%	51	18.9%	38	24.1%	
		Satisfied	140	32.7%	91	33.7%	49	31.0%	
		Very satisfied	101	23.6%	62	23.0%	39	24.7%	
	26. How often do you have negative feelings such as blue mood, despair, anxiety, depression?	Never	31	7.2%	18	6.7%	13	8.2%	0.072
		Seldom	94	22.0%	58	21.5%	36	22.8%	
		Quite often	126	29.4%	76	28.1%	50	31.6%	
		Very often	124	29.0%	75	27.8%	49	31.0%	
		Always	53	12.4%	43	15.9%	10	6.3%	
Social	20. How satisfied are you with your personal relationships?	Very dissatisfied	52	12.1%	29	10.7%	23	14.6%	0.194
		Dissatisfied	59	13.8%	34	12.6%	25	15.8%	
		Neither satisfied nor dissatisfied	100	23.4%	62	23.0%	38	24.1%	
		Satisfied	129	30.1%	92	34.1%	37	23.4%	
		Very satisfied	88	20.6%	53	19.6%	35	22.2%	
	21. How satisfied are you with your sex life?	Very dissatisfied	70	16.4%	38	14.1%	32	20.3%	0.002
		Dissatisfied	70	16.4%	34	12.6%	36	22.8%	
		Neither satisfied nor dissatisfied	126	29.4%	80	29.6%	46	29.1%	
		Satisfied	108	25.2%	82	30.4%	26	16.5%	

		Very satisfied	54	12.6%	36	13.3%	18	11.4%	
	22. How satisfied are you with the support you get from your friends?	Very dissatisfied	72	16.8%	47	17.4%	25	15.8%	
		Dissatisfied	58	13.6%	35	13.0%	23	14.6%	
		Neither satisfied nor dissatisfied	107	25.0%	54	20.0%	53	33.5%	0.011
		Satisfied	113	26.4%	83	30.7%	30	19.0%	
		Very satisfied	78	18.2%	51	18.9%	27	17.1%	
	8. How safe do you feel in your daily life?	Not at all	59	13.8%	37	13.7%	22	13.9%	
		A little	6	1.4%	5	1.9%	1	0.6%	
		A moderate amount	133	31.1%	82	30.4%	51	32.3%	0.105
		Very much	102	23.8%	74	27.4%	28	17.7%	
		Extremely	128	29.9%	72	26.7%	56	35.4%	
	9. How healthy is your physical environment?	Not at all	40	9.3%	26	9.6%	14	8.9%	
		A little	6	1.4%	4	1.5%	2	1.3%	
		A moderate amount	160	37.4%	96	35.6%	64	40.5%	0.767
		Very much	120	28.0%	81	30.0%	39	24.7%	
		Extremely	102	23.8%	63	23.3%	39	24.7%	
	12. Have you enough money to meet your needs?	Not at all	75	17.5%	46	17.0%	29	18.4%	
		A little	79	18.5%	47	17.4%	32	20.3%	
		Moderately	103	24.1%	68	25.2%	35	22.2%	0.893
		Mostly	82	19.2%	51	18.9%	31	19.6%	
		Completely	89	20.8%	58	21.5%	31	19.6%	
	13. How available to you is the information that you need in your day-to-day life?	Not at all	33	7.7%	21	7.8%	12	7.6%	
		A little	83	19.4%	41	15.2%	42	26.6%	
		Moderately	112	26.2%	78	28.9%	34	21.5%	0.038
		Mostly	107	25.0%	66	24.4%	41	25.9%	
		Completely	93	21.7%	64	23.7%	290	18.4%	
Environmental	14. To what extent do you have the opportunity for leisure activities?	Not at all	76	17.8%	46	17.0%	30	19.0%	
		A little	133	31.1%	78	28.9%	55	34.8%	
		Moderately	100	23.4%	63	23.3%	37	23.4%	0.450
		Mostly	77	18.0%	53	19.6%	24	15.2%	
		Completely	42	9.8%	30	11.1%	12	7.6%	
	23. How satisfied are you with the conditions of your living place?	Very dissatisfied	51	11.9%	36	13.3%	15	9.5%	
		Dissatisfied	42	9.8%	25	9.3%	17	10.8%	
		Neither satisfied nor dissatisfied	95	22.2%	59	21.9%	36	22.8%	0.612
		Satisfied	128	29.9%	84	31.1%	44	27.8%	
		Very satisfied	112	26.2%	66	24.4%	46	29.1%	
	24. How satisfied are you with your access to health services?	Very dissatisfied	58	13.6%	39	14.4%	19	12.0%	
		Dissatisfied	65	15.2%	41	15.2%	24	15.2%	
		Neither satisfied nor dissatisfied	85	19.9%	45	16.7%	40	25.3%	0.152
		Satisfied	124	29.0%	77	28.5%	47	29.7%	
		Very satisfied	96	22.4%	68	25.2%	28	17.7%	
	25. How satisfied are you with your transport?	Very dissatisfied	41	9.6%	21	7.8%	20	12.7%	
		Dissatisfied	50	11.7%	33	12.2%	17	10.8%	
		Neither satisfied nor dissatisfied	70	16.4%	46	17.0%	24	15.2%	0.432
		Satisfied	137	32.0%	91	33.7%	46	29.1%	
		Very satisfied	130	30.4%	79	29.3%	51	32.3%	
